# Crystal structure of bis­(3,3-dimethyl-2-oxobut­yl)di­phenyl­phospho­nium bromide chloro­form monosolvate

**DOI:** 10.1107/S205698901500763X

**Published:** 2015-04-25

**Authors:** Alyssa A. Kulesza, Richard J. Staples, Shannon M. Biros

**Affiliations:** a Department of Chemistry, Grand Valley State University, 1 Campus Dr., Allendale, MI 49401, USA; b Center for Crystallographic Research, Department of Chemistry, Michigan State University, 578 S. Shaw Lane, East Lansing, MI 48824, USA

**Keywords:** crystal structure, phospho­nium bromide salt, isopropoxydi­phenyl­phosphane, bromo­pinacolone, Arbuzov reaction

## Abstract

In the title salt solvate, C_24_H_32_O_2_P^+^·Br^−^·CHCl_3_, the P atom has a distorted tetra­hedral geometry, and the planes of the phenyl rings form a dihedral angle of 71.86 (14)° with one another. The bromide anion is disordered and was modelled over three positions (occupancy ratio 0.50:0.35:0.15). The crystal also contains one disordered chloro­form solvent mol­ecule that was modeled over three positions (occupancy ratio 0.50:0.35:0.15). Weak inter­molecular inter­actions (C—H⋯Br and C—H⋯O) exist between the complex cation and the bromide anion fragments. The resulting supramolecular structure is an oval-shaped arrangement of phosphonium salt molecules that surround the disordered bromide anion.

## Related literature   

The title compound was synthesized using an Arbuzov reaction, as described by Schuster *et al.* (2009 ▸). The Cambridge Structural Database (CSD, Version 5.36, November 2014; Groom & Allen, 2014 ▸) contains four structures of acyclic tetra­valent phospho­nium salts where the P atom is bonded to two phenyl rings and two β-carbonyl groups. In each structure, the phospho­nium salt is coordinated to a silver(I) (Vicente *et al.*, 1989 ▸) or palladium(II) (Vicente *et al.*, 1990 ▸) metal center *via* the carbon α to the P atom.
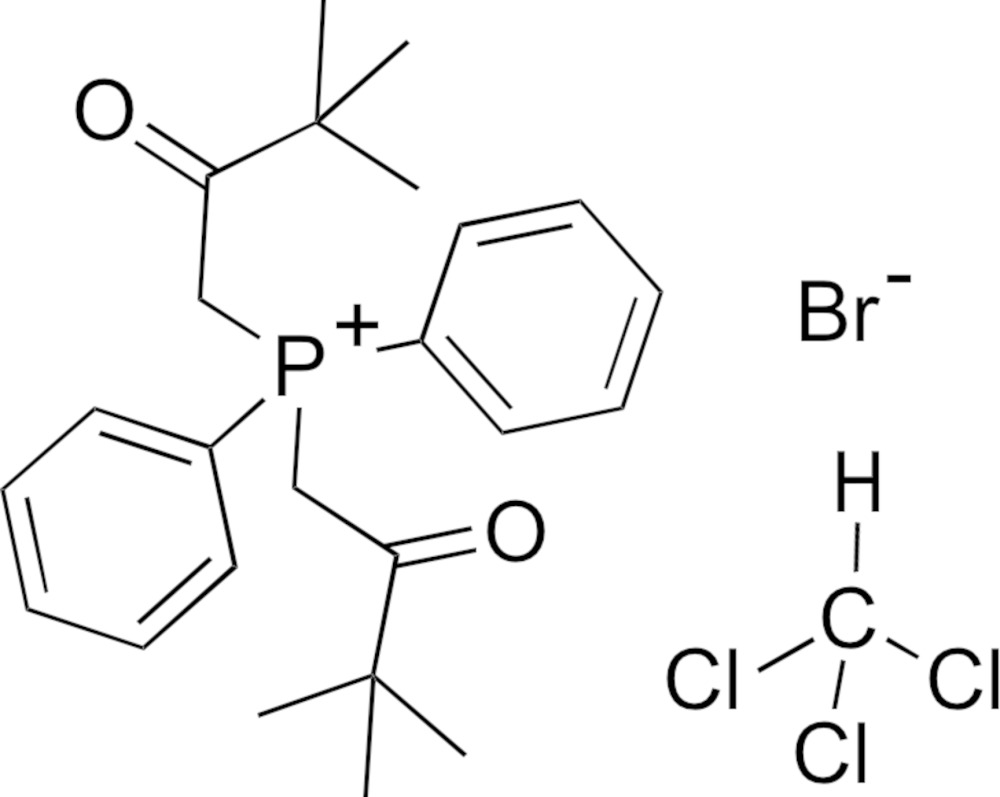


## Experimental   

### Crystal data  

C_24_H_32_O_2_P^+^·Br^−^·CHCl_3_
*M* *_r_* = 582.74
Monoclinic, 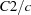

*a* = 23.6380 (18) Å
*b* = 13.6273 (10) Å
*c* = 18.1817 (13) Å
β = 108.702 (1)°
*V* = 5547.5 (7) Å^3^
*Z* = 8
Mo *K*α radiation
μ = 1.85 mm^−1^
*T* = 173 K
0.34 × 0.15 × 0.09 mm

### Data collection  

Bruker SMART APEX CCD area-detector diffractometer
Absorption correction: multi-scan (*SADABS*; Bruker, 2013 ▸) *T*_min_ = 0.667, *T*_max_ = 0.745
25767 measured reflections
5090 independent reflections
3862 reflections with *I* > 2σ(*I*)
*R*_int_ = 0.040

### Refinement  

*R*[*F* ^2^ > 2σ(*F* ^2^)] = 0.064
*wR*(*F* ^2^) = 0.206
*S* = 1.04
5090 reflections
373 parameters
66 restraints
H-atom parameters constrained
Δρ_max_ = 1.30 e Å^−3^
Δρ_min_ = −1.10 e Å^−3^

### 

Data collection: *APEX2* (Bruker, 2013 ▸); cell refinement: *SAINT* (Bruker, 2013 ▸); data reduction: *SAINT*; program(s) used to solve structure: *SHELXS97* (Sheldrick, 2008 ▸); program(s) used to refine structure: *SHELXL2014* (Sheldrick, 2015 ▸); molecular graphics: *CrystalMaker* (Palmer, 2007 ▸); software used to prepare material for publication: *OLEX2* (Dolomanov *et al.*, 2009 ▸; Bourhis *et al.*, 2015 ▸).

## Supplementary Material

Crystal structure: contains datablock(s) I. DOI: 10.1107/S205698901500763X/pk2549sup1.cif

Structure factors: contains datablock(s) I. DOI: 10.1107/S205698901500763X/pk2549Isup2.hkl

Supporting information file. DOI: 10.1107/S205698901500763X/pk2549Isup3.cml

**Fig. 1.**
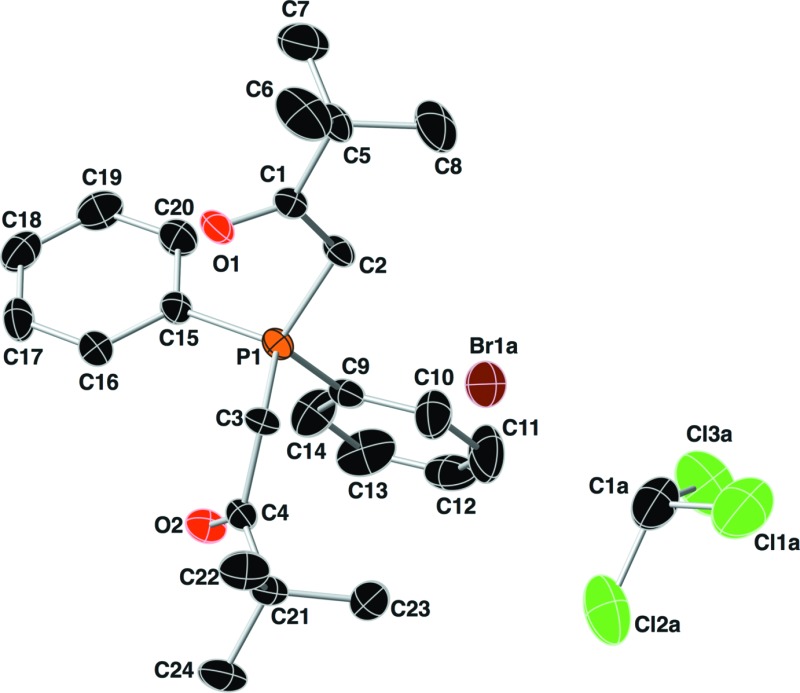
Structure of the asymmetric unit of I along with the atom numbering scheme. This drawing is done with 50% probability ellipsoids using standard CPK colors; only one position of the disordered bromide ion and chloroform molecule is shown, and all hydrogen atoms have been omitted for clarity.

I . DOI: 10.1107/S205698901500763X/pk2549fig1.tif
Structure of the asymmetric unit of **I** along with the atom numbering scheme. This drawing is done with 50% probability ellipsoids using standard CPK colors; only one position of the disordered bromide ion and chloro­form mol­ecule is shown, and all hydrogen atoms have been omitted for clarity.

**Fig. 2.**
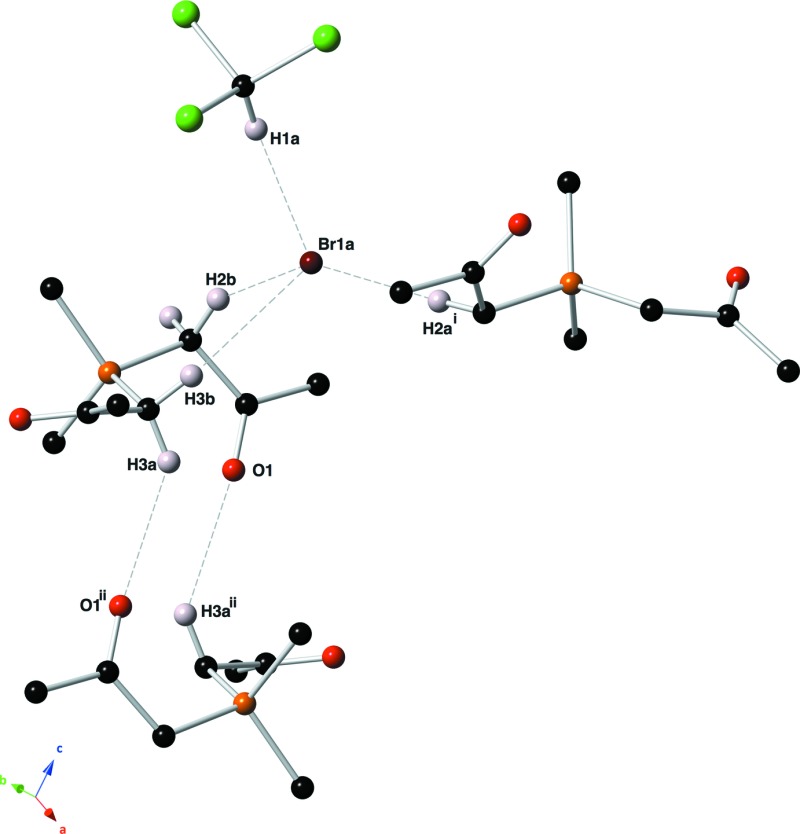
Weak intermolecular interactions (denoted with dashed lines) found throughout the crystal lattice of the title compound (Table 1). Only the major position of the disordered fragments are shown. The aryl rings, tert-butyl groups, and all hydrogen atoms not involved in these interactions have been omitted for clarity. This drawing is done as a ball and stick with standard CPK colors. Symmetry codes: (i) 3/2 - x, -1/2 + y, 3/2 - z; (ii) 3/2 - x, 1/2 - y, 1 - z.

Click here for additional data file.tert x y z x y z . DOI: 10.1107/S205698901500763X/pk2549fig2.tif
Weak inter­molecular inter­actions (denoted with dashed lines) found throughout the crystal lattice of the title compound (Table 1). Only the major position of the disordered fragments are shown. The aryl rings, *tert*-butyl groups, and all hydrogen atoms not involved in these inter­actions have been omitted for clarity. This drawing is done as a ball and stick with standard CPK colors. Symmetry codes: (i) 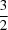 − *x*, −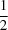 + *y*, 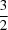 − *z*; (ii) 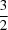 − *x*, 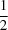 − *y*, 1 − *z*.

CCDC reference: 1060276

Additional supporting information:  crystallographic information; 3D view; checkCIF report

## Figures and Tables

**Table 1:** Hydrogen-bond geometry (, )

*D*H*A*	*D*H	H*A*	*D* *A*	*D*H*A*
C2H2*A*Br1*B* ^i^	0.99	2.85	3.840(9)	174
C2H2*A*Br1*C* ^i^	0.99	2.75	3.712(6)	164
C2H2*A*Br1*A* ^i^	0.99	2.95	3.941(7)	177
C2H2*B*Br1*B*	0.99	2.95	3.767(9)	141
C2H2*B*Br1*C*	0.99	2.97	3.717(6)	133
C2H2*B*Br1*A*	0.99	2.97	3.831(7)	145
C3H3*B*Br1*B*	0.99	2.78	3.740(9)	163
C3H3*B*Br1*C*	0.99	2.70	3.644(6)	159
C3H3*B*Br1*A*	0.99	2.86	3.816(7)	164
C16H16O1^ii^	0.95	2.43	3.287(5)	150
C1*A*H1*A*Br1*A*	1.00	2.59	3.527(9)	156

Symmetry codes: (i) 
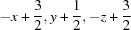
; (ii) 
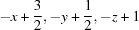
.

## supplementary crystallographic information

### S1. Structural commentary

The molecular structure of I is shown in Figure 1, along with the atom numbering scheme. Compound I crystallized in the monoclinic space group *C*2/c, and the asymmetric unit contains the entire molecule along with one disordered bromide anion and one disordered molecule of chloro­form. Atom P1 has a distorted tetra­hedral geometry with C—P—C bond angles ranging from 106.37 (18) to 113.10 (18)°.

### S2. Supra­molecular features

Weak inter­molecular C—H···Br and C—H···O inter­actions can be found throughout the crystal lattice (Table 1). CH···O hydrogen bonds (2.43 Å) exist between the carbonyl oxygen O1 and an aromatic hydrogen H16 (Figure 2). The bromide ion is engaged in a variety of weak inter­actions with nearby hydrogen atoms, with inter­atomic H···Br distances ranging from 2.70 to 2.97 Å. In the fragment that has the highest occupancy ratio (50%), a weak CH···Br inter­action is also present between the chloro­form molecule and bromide ion. Based on the amount of disorder present in this structure, it is clear these inter­molecular inter­actions are quite weak in nature.

### S3. Synthesis and crystallization

The title compound was prepared via an Arbuzov reaction between bromo­pinacolone and an excess of *iso*-propoxydi­phenyl phosphane following the procedure of Schuster *et al.* (2009). ^1^H NMR (CDCl_3_, 300 MHz), δ 7.95-7.27 (m, 10H), 5.47 (d, *J_HP_* =6 Hz, 4H), 3.6 (d, J=2 Hz, 2H), 1.1 (s, 18 H). Crystals of I suitable for analysis by X-Ray diffraction were grown from vapor diffusion of ethyl acetate into a solution of CHCl_3_ containing Tb(NO_3_)_3_.

### S4. Refinement

Crystal data, data collection and structure refinement details are summarized in Table 1. The positions of all hydrogen atoms were calculated geometrically and refined to ride on their parent atoms, with *U*_iso_(H) = 1.2*U*_eq_(C) for methine, methyl­ene and aryl groups, and *U*_iso_(H) = 1.5*U*_eq_(C) for methyl groups.

The disordered bromide ion was modelled over three positions with a 50:35:15 occupancy ratio. An EADP instruction was included in the refinement to constrain the thermal ellipoids of these fragments to the same size and shape. The disordered chloro­form molecule was also modelled in three parts with a 50:35:15 occupancy ratio. To restrain the CHCl_3_ geometry to accepted values, each C—Cl bond length was restrained with a DFIX instruction to 1.78 Å, and the Cl—Cl inter­atomic bond distances were restrained with a DANG instruction to 2.87 Å. Finally, each chlofororm fragment was treated with SIMU and DELU instructions to ensure uniform thermal ellipsoids.

### Crystal data

**Table:**

C_24_H_32_O_2_P^+^·Br^−^·CHCl_3_	*F*(000) = 2400
*M**_r_* = 582.74	*D*_x_ = 1.395 Mg m^−^^3^
Monoclinic, *C*2/*c*	Mo *K*α radiation, λ = 0.71073 Å
*a* = 23.6380 (18) Å	Cell parameters from 9180 reflections
*b* = 13.6273 (10) Å	θ = 2.3–25.3°
*c* = 18.1817 (13) Å	µ = 1.85 mm^−^^1^
β = 108.702 (1)°	*T* = 173 K
*V* = 5547.5 (7) Å^3^	Plate, colourless
*Z* = 8	0.34 × 0.15 × 0.09 mm

### Data collection

**Table:**

Bruker SMART APEX CCD area-detector diffractometer	5090 independent reflections
Radiation source: sealed tube	3862 reflections with *I* > 2σ(*I*)
Graphite monochromator	*R*_int_ = 0.040
Detector resolution: 8 pixels mm^-1^	θ_max_ = 25.4°, θ_min_ = 1.8°
ω and φ scans	*h* = −28→28
Absorption correction: multi-scan (*SADABS*; Bruker, 2013)	*k* = −15→16
*T*_min_ = 0.667, *T*_max_ = 0.745	*l* = −19→21
25767 measured reflections	

### Refinement

**Table:**

Refinement on *F*^2^	Primary atom site location: structure-invariant direct methods
Least-squares matrix: full	Hydrogen site location: inferred from neighbouring sites
*R*[*F*^2^ > 2σ(*F*^2^)] = 0.064	H-atom parameters constrained
*wR*(*F*^2^) = 0.206	*w* = 1/[σ^2^(*F*_o_^2^) + (0.1214*P*)^2^ + 22.4904*P*] where *P* = (*F*_o_^2^ + 2*F*_c_^2^)/3
*S* = 1.04	(Δ/σ)_max_ = 0.001
5090 reflections	Δρ_max_ = 1.30 e Å^−^^3^
373 parameters	Δρ_min_ = −1.09 e Å^−^^3^
66 restraints	

### Special details

**Table:**

Experimental. SADABS-2012/1 (Bruker, 2013) was used for absorption correction. wR2(int) was 0.0613 before and 0.0433 after correction. The Ratio of minimum to maximum transmission is 0.8956. The λ/2 correction factor is 0.0015.
Geometry. All e.s.d.'s (except the e.s.d. in the dihedral angle between two l.s. planes) are estimated using the full covariance matrix. The cell e.s.d.'s are taken into account individually in the estimation of e.s.d.'s in distances, angles and torsion angles; correlations between e.s.d.'s in cell parameters are only used when they are defined by crystal symmetry. An approximate (isotropic) treatment of cell e.s.d.'s is used for estimating e.s.d.'s involving l.s. planes.

### Fractional atomic coordinates and isotropic or equivalent isotropic displacement parameters (Å^2^)

**Table:**

	*x*	*y*	*z*	*U*_iso_*/*U*_eq_	Occ. (<1)
P1	0.69139 (4)	0.39763 (7)	0.58769 (6)	0.0214 (3)	
O1	0.80869 (13)	0.3191 (2)	0.60430 (17)	0.0309 (7)	
O2	0.58476 (13)	0.3136 (2)	0.46946 (18)	0.0305 (7)	
C1	0.81047 (17)	0.3556 (3)	0.6659 (2)	0.0243 (9)	
C2	0.75533 (17)	0.4046 (3)	0.6746 (2)	0.0254 (9)	
H2A	0.7644	0.4745	0.6883	0.030*	
H2B	0.7452	0.3731	0.7178	0.030*	
C3	0.67433 (17)	0.2690 (3)	0.5678 (2)	0.0233 (8)	
H3A	0.7025	0.2410	0.5432	0.028*	
H3B	0.6805	0.2342	0.6176	0.028*	
C4	0.61055 (17)	0.2511 (3)	0.5150 (2)	0.0237 (8)	
C5	0.86786 (19)	0.3561 (3)	0.7359 (2)	0.0306 (10)	
C6	0.9052 (3)	0.2661 (5)	0.7318 (4)	0.0618 (17)	
H6A	0.8827	0.2065	0.7342	0.093*	
H6B	0.9424	0.2670	0.7757	0.093*	
H6C	0.9145	0.2671	0.6830	0.093*	
C7	0.9016 (3)	0.4498 (5)	0.7256 (4)	0.0568 (15)	
H7A	0.8757	0.5071	0.7213	0.085*	
H7B	0.9129	0.4440	0.6784	0.085*	
H7C	0.9377	0.4577	0.7707	0.085*	
C8	0.8561 (3)	0.3630 (6)	0.8130 (3)	0.0650 (18)	
H8A	0.8354	0.4247	0.8152	0.098*	
H8B	0.8942	0.3613	0.8555	0.098*	
H8C	0.8313	0.3076	0.8181	0.098*	
C9	0.63073 (18)	0.4559 (3)	0.6102 (2)	0.0273 (9)	
C10	0.6175 (2)	0.4284 (5)	0.6759 (3)	0.0496 (14)	
H10	0.6406	0.3793	0.7095	0.060*	
C11	0.5699 (3)	0.4733 (6)	0.6925 (4)	0.0682 (19)	
H11	0.5607	0.4554	0.7379	0.082*	
C12	0.5360 (2)	0.5440 (4)	0.6428 (4)	0.0560 (16)	
H12	0.5036	0.5745	0.6542	0.067*	
C13	0.5489 (2)	0.5695 (4)	0.5781 (4)	0.0557 (16)	
H13	0.5254	0.6176	0.5440	0.067*	
C14	0.5961 (2)	0.5261 (4)	0.5616 (3)	0.0458 (13)	
H14	0.6049	0.5447	0.5160	0.055*	
C15	0.70589 (17)	0.4604 (3)	0.5093 (2)	0.0240 (8)	
C16	0.69455 (19)	0.4162 (3)	0.4366 (2)	0.0291 (9)	
H16	0.6794	0.3512	0.4281	0.035*	
C17	0.7055 (2)	0.4676 (4)	0.3773 (3)	0.0365 (11)	
H17	0.6985	0.4374	0.3281	0.044*	
C18	0.7268 (2)	0.5631 (4)	0.3890 (3)	0.0402 (11)	
H18	0.7330	0.5989	0.3474	0.048*	
C19	0.7389 (2)	0.6061 (4)	0.4609 (3)	0.0411 (12)	
H19	0.7543	0.6710	0.4689	0.049*	
C20	0.7288 (2)	0.5556 (3)	0.5220 (3)	0.0353 (10)	
H20	0.7374	0.5854	0.5716	0.042*	
C21	0.58202 (18)	0.1535 (3)	0.5240 (3)	0.0285 (9)	
C22	0.6255 (2)	0.0693 (4)	0.5296 (3)	0.0435 (12)	
H22A	0.6409	0.0723	0.4856	0.065*	
H22B	0.6049	0.0067	0.5285	0.065*	
H22C	0.6588	0.0747	0.5783	0.065*	
C23	0.5652 (2)	0.1610 (4)	0.5987 (3)	0.0456 (12)	
H23A	0.6016	0.1661	0.6435	0.068*	
H23B	0.5428	0.1024	0.6040	0.068*	
H23C	0.5404	0.2194	0.5962	0.068*	
C24	0.5254 (2)	0.1393 (4)	0.4537 (3)	0.0427 (12)	
H24A	0.4981	0.1943	0.4506	0.064*	
H24B	0.5058	0.0778	0.4596	0.064*	
H24C	0.5364	0.1367	0.4061	0.064*	
Cl1A	0.61055 (18)	0.1585 (4)	0.9366 (2)	0.1062 (18)	0.5
Cl2A	0.53420 (17)	0.1851 (3)	0.77966 (18)	0.0794 (11)	0.5
Cl3A	0.5717 (5)	0.3487 (3)	0.8797 (5)	0.094 (3)	0.5
C1A	0.5960 (3)	0.2348 (4)	0.8538 (3)	0.061 (3)	0.5
H1A	0.6319	0.2423	0.8365	0.074*	0.5
Cl1B	0.5809 (3)	0.1342 (3)	0.8669 (5)	0.0963 (18)	0.35
Cl2B	0.6295 (4)	0.2351 (6)	1.0065 (4)	0.061 (2)	0.35
Cl3B	0.5721 (5)	0.3442 (4)	0.8666 (6)	0.096 (5)	0.35
C1B	0.6172 (5)	0.2428 (4)	0.9116 (10)	0.078 (4)	0.35
H1B	0.6565	0.2479	0.9022	0.093*	0.35
Cl1C	0.6129 (8)	0.2150 (13)	0.9961 (6)	0.060 (5)	0.15
Cl2C	0.5472 (5)	0.3486 (8)	0.8770 (10)	0.045 (3)	0.15
Cl3C	0.6339 (3)	0.2186 (5)	0.8493 (5)	0.0368 (17)	0.15
C1C	0.5777 (5)	0.2284 (8)	0.8944 (7)	0.051 (5)	0.15
H1C	0.5460	0.1775	0.8740	0.061*	0.15
Br1B	0.7228 (3)	0.1780 (6)	0.7727 (5)	0.0420 (5)	0.35
Br1C	0.7376 (2)	0.1642 (3)	0.7613 (3)	0.0420 (5)	0.15
Br1A	0.7134 (2)	0.1856 (4)	0.7786 (3)	0.0420 (5)	0.5

### Atomic displacement parameters (Å^2^)

**Table:**

	*U*^11^	*U*^22^	*U*^33^	*U*^12^	*U*^13^	*U*^23^
P1	0.0198 (5)	0.0230 (5)	0.0183 (5)	0.0017 (4)	0.0015 (4)	−0.0006 (4)
O1	0.0306 (16)	0.0357 (17)	0.0222 (16)	0.0038 (13)	0.0026 (12)	−0.0079 (13)
O2	0.0246 (15)	0.0291 (16)	0.0307 (17)	0.0039 (12)	−0.0011 (13)	0.0053 (13)
C1	0.023 (2)	0.023 (2)	0.024 (2)	0.0004 (16)	0.0030 (16)	0.0006 (17)
C2	0.0222 (19)	0.030 (2)	0.0189 (19)	−0.0005 (16)	0.0000 (15)	−0.0048 (16)
C3	0.0196 (19)	0.0214 (19)	0.024 (2)	−0.0006 (15)	0.0004 (16)	−0.0012 (16)
C4	0.0218 (19)	0.026 (2)	0.022 (2)	0.0043 (16)	0.0049 (16)	−0.0032 (17)
C5	0.026 (2)	0.039 (2)	0.021 (2)	0.0037 (18)	−0.0012 (17)	−0.0057 (18)
C6	0.042 (3)	0.072 (4)	0.054 (4)	0.024 (3)	−0.009 (3)	−0.006 (3)
C7	0.042 (3)	0.064 (4)	0.057 (4)	−0.012 (3)	0.006 (3)	−0.011 (3)
C8	0.036 (3)	0.120 (6)	0.029 (3)	0.009 (3)	−0.004 (2)	−0.007 (3)
C9	0.024 (2)	0.025 (2)	0.029 (2)	−0.0022 (16)	0.0042 (17)	−0.0087 (17)
C10	0.037 (3)	0.078 (4)	0.034 (3)	0.015 (3)	0.013 (2)	0.008 (3)
C11	0.049 (3)	0.122 (6)	0.042 (3)	0.011 (4)	0.025 (3)	−0.014 (4)
C12	0.033 (3)	0.059 (4)	0.078 (4)	0.004 (3)	0.021 (3)	−0.032 (3)
C13	0.038 (3)	0.039 (3)	0.092 (5)	0.016 (2)	0.025 (3)	0.002 (3)
C14	0.043 (3)	0.042 (3)	0.059 (3)	0.014 (2)	0.025 (3)	0.011 (2)
C15	0.0220 (19)	0.027 (2)	0.021 (2)	0.0041 (16)	0.0036 (16)	0.0037 (16)
C16	0.030 (2)	0.029 (2)	0.027 (2)	0.0009 (17)	0.0077 (18)	−0.0013 (18)
C17	0.040 (3)	0.048 (3)	0.022 (2)	0.006 (2)	0.0098 (19)	0.004 (2)
C18	0.037 (3)	0.045 (3)	0.043 (3)	0.005 (2)	0.017 (2)	0.016 (2)
C19	0.041 (3)	0.030 (2)	0.055 (3)	−0.005 (2)	0.018 (2)	0.005 (2)
C20	0.037 (2)	0.033 (2)	0.035 (3)	−0.0035 (19)	0.010 (2)	−0.006 (2)
C21	0.023 (2)	0.029 (2)	0.029 (2)	−0.0031 (17)	0.0013 (17)	0.0030 (18)
C22	0.040 (3)	0.030 (2)	0.055 (3)	0.000 (2)	0.008 (2)	0.004 (2)
C23	0.041 (3)	0.058 (3)	0.039 (3)	−0.010 (2)	0.014 (2)	0.003 (2)
C24	0.034 (3)	0.040 (3)	0.043 (3)	−0.012 (2)	−0.004 (2)	0.002 (2)
Cl1A	0.066 (2)	0.165 (5)	0.081 (3)	0.035 (3)	0.014 (2)	0.074 (3)
Cl2A	0.096 (3)	0.090 (3)	0.0519 (19)	0.032 (2)	0.0233 (18)	−0.0061 (17)
Cl3A	0.105 (8)	0.092 (6)	0.077 (4)	0.007 (5)	0.017 (5)	−0.015 (4)
C1A	0.057 (8)	0.071 (9)	0.061 (8)	0.013 (7)	0.026 (7)	0.022 (7)
Cl1B	0.082 (4)	0.084 (4)	0.119 (5)	−0.001 (3)	0.028 (4)	−0.001 (4)
Cl2B	0.062 (4)	0.056 (5)	0.069 (3)	0.008 (3)	0.029 (3)	0.020 (3)
Cl3B	0.078 (8)	0.092 (7)	0.094 (7)	−0.011 (6)	−0.005 (5)	0.041 (5)
C1B	0.076 (9)	0.075 (8)	0.082 (7)	−0.009 (7)	0.025 (8)	0.020 (8)
Cl1C	0.063 (10)	0.029 (6)	0.099 (10)	0.001 (7)	0.039 (7)	0.023 (6)
Cl2C	0.052 (7)	0.028 (4)	0.047 (6)	0.008 (4)	0.006 (6)	0.004 (4)
Cl3C	0.033 (4)	0.034 (4)	0.049 (4)	−0.002 (3)	0.022 (3)	−0.017 (3)
C1C	0.048 (10)	0.031 (9)	0.081 (10)	0.004 (9)	0.033 (8)	−0.001 (10)
Br1B	0.0474 (16)	0.0398 (10)	0.0380 (10)	0.0058 (9)	0.0124 (8)	0.0082 (7)
Br1C	0.0474 (16)	0.0398 (10)	0.0380 (10)	0.0058 (9)	0.0124 (8)	0.0082 (7)
Br1A	0.0474 (16)	0.0398 (10)	0.0380 (10)	0.0058 (9)	0.0124 (8)	0.0082 (7)

### Geometric parameters (Å, º)

**Table:**

P1—C2	1.805 (4)	C15—C16	1.398 (6)
P1—C3	1.809 (4)	C15—C20	1.397 (6)
P1—C9	1.798 (4)	C16—H16	0.9500
P1—C15	1.786 (4)	C16—C17	1.379 (6)
O1—C1	1.214 (5)	C17—H17	0.9500
O2—C4	1.208 (5)	C17—C18	1.387 (7)
C1—C2	1.517 (6)	C18—H18	0.9500
C1—C5	1.534 (6)	C18—C19	1.376 (7)
C2—H2A	0.9900	C19—H19	0.9500
C2—H2B	0.9900	C19—C20	1.391 (7)
C3—H3A	0.9900	C20—H20	0.9500
C3—H3B	0.9900	C21—C22	1.522 (6)
C3—C4	1.526 (5)	C21—C23	1.536 (7)
C4—C21	1.523 (6)	C21—C24	1.537 (6)
C5—C6	1.526 (7)	C22—H22A	0.9800
C5—C7	1.549 (8)	C22—H22B	0.9800
C5—C8	1.517 (7)	C22—H22C	0.9800
C6—H6A	0.9800	C23—H23A	0.9800
C6—H6B	0.9800	C23—H23B	0.9800
C6—H6C	0.9800	C23—H23C	0.9800
C7—H7A	0.9800	C24—H24A	0.9800
C7—H7B	0.9800	C24—H24B	0.9800
C7—H7C	0.9800	C24—H24C	0.9800
C8—H8A	0.9800	Cl1A—C1A	1.770 (5)
C8—H8B	0.9800	Cl2A—Cl2A^i^	1.631 (7)
C8—H8C	0.9800	Cl2A—C1A	1.774 (5)
C9—C10	1.379 (7)	Cl3A—C1A	1.772 (5)
C9—C14	1.380 (7)	C1A—H1A	1.0000
C10—H10	0.9500	Cl1B—C1B	1.772 (5)
C10—C11	1.397 (8)	Cl2B—C1B	1.66 (2)
C11—H11	0.9500	Cl3B—C1B	1.776 (5)
C11—C12	1.386 (10)	C1B—H1B	1.0000
C12—H12	0.9500	Cl1C—C1C	1.778 (5)
C12—C13	1.351 (9)	Cl2C—C1C	1.777 (5)
C13—H13	0.9500	Cl3C—C1C	1.776 (5)
C13—C14	1.378 (7)	C1C—H1C	1.0000
C14—H14	0.9500		
			
C2—P1—C3	107.24 (19)	C13—C14—C9	120.9 (5)
C9—P1—C2	106.40 (19)	C13—C14—H14	119.6
C9—P1—C3	109.30 (19)	C16—C15—P1	121.4 (3)
C15—P1—C2	110.60 (19)	C20—C15—P1	118.4 (3)
C15—P1—C3	113.08 (19)	C20—C15—C16	120.2 (4)
C15—P1—C9	110.0 (2)	C15—C16—H16	120.2
O1—C1—C2	120.0 (4)	C17—C16—C15	119.6 (4)
O1—C1—C5	121.8 (4)	C17—C16—H16	120.2
C2—C1—C5	118.2 (3)	C16—C17—H17	119.8
P1—C2—H2A	109.0	C16—C17—C18	120.4 (4)
P1—C2—H2B	109.0	C18—C17—H17	119.8
C1—C2—P1	113.1 (3)	C17—C18—H18	120.0
C1—C2—H2A	109.0	C19—C18—C17	120.0 (4)
C1—C2—H2B	109.0	C19—C18—H18	120.0
H2A—C2—H2B	107.8	C18—C19—H19	119.6
P1—C3—H3A	108.9	C18—C19—C20	120.8 (4)
P1—C3—H3B	108.9	C20—C19—H19	119.6
H3A—C3—H3B	107.8	C15—C20—H20	120.5
C4—C3—P1	113.1 (3)	C19—C20—C15	118.9 (4)
C4—C3—H3A	108.9	C19—C20—H20	120.5
C4—C3—H3B	108.9	C4—C21—C23	106.6 (4)
O2—C4—C3	119.9 (4)	C4—C21—C24	108.5 (4)
O2—C4—C21	123.1 (4)	C22—C21—C4	110.6 (3)
C21—C4—C3	117.0 (3)	C22—C21—C23	110.6 (4)
C1—C5—C7	104.9 (4)	C22—C21—C24	110.5 (4)
C6—C5—C1	109.2 (4)	C23—C21—C24	109.9 (4)
C6—C5—C7	109.2 (5)	C21—C22—H22A	109.5
C8—C5—C1	113.1 (4)	C21—C22—H22B	109.5
C8—C5—C6	112.0 (5)	C21—C22—H22C	109.5
C8—C5—C7	108.3 (5)	H22A—C22—H22B	109.5
C5—C6—H6A	109.5	H22A—C22—H22C	109.5
C5—C6—H6B	109.5	H22B—C22—H22C	109.5
C5—C6—H6C	109.5	C21—C23—H23A	109.5
H6A—C6—H6B	109.5	C21—C23—H23B	109.5
H6A—C6—H6C	109.5	C21—C23—H23C	109.5
H6B—C6—H6C	109.5	H23A—C23—H23B	109.5
C5—C7—H7A	109.5	H23A—C23—H23C	109.5
C5—C7—H7B	109.5	H23B—C23—H23C	109.5
C5—C7—H7C	109.5	C21—C24—H24A	109.5
H7A—C7—H7B	109.5	C21—C24—H24B	109.5
H7A—C7—H7C	109.5	C21—C24—H24C	109.5
H7B—C7—H7C	109.5	H24A—C24—H24B	109.5
C5—C8—H8A	109.5	H24A—C24—H24C	109.5
C5—C8—H8B	109.5	H24B—C24—H24C	109.5
C5—C8—H8C	109.5	Cl2A^i^—Cl2A—C1A	154.5 (3)
H8A—C8—H8B	109.5	Cl1A—C1A—Cl2A	108.1 (4)
H8A—C8—H8C	109.5	Cl1A—C1A—Cl3A	106.2 (4)
H8B—C8—H8C	109.5	Cl1A—C1A—H1A	112.0
C10—C9—P1	119.7 (4)	Cl2A—C1A—H1A	112.0
C10—C9—C14	119.4 (4)	Cl3A—C1A—Cl2A	106.2 (4)
C14—C9—P1	120.8 (4)	Cl3A—C1A—H1A	112.0
C9—C10—H10	120.4	Cl1B—C1B—Cl3B	107.9 (4)
C9—C10—C11	119.3 (5)	Cl1B—C1B—H1B	108.7
C11—C10—H10	120.4	Cl2B—C1B—Cl1B	108.7 (10)
C10—C11—H11	120.0	Cl2B—C1B—Cl3B	114.0 (10)
C12—C11—C10	120.1 (6)	Cl2B—C1B—H1B	108.7
C12—C11—H11	120.0	Cl3B—C1B—H1B	108.7
C11—C12—H12	120.0	Cl1C—C1C—H1C	111.1
C13—C12—C11	120.1 (5)	Cl2C—C1C—Cl1C	107.9 (4)
C13—C12—H12	120.0	Cl2C—C1C—H1C	111.1
C12—C13—H13	119.9	Cl3C—C1C—Cl1C	107.6 (4)
C12—C13—C14	120.3 (5)	Cl3C—C1C—Cl2C	107.9 (4)
C14—C13—H13	119.9	Cl3C—C1C—H1C	111.1
C9—C14—H14	119.6		
			
P1—C3—C4—O2	26.8 (5)	C3—C4—C21—C22	−44.8 (5)
P1—C3—C4—C21	−152.1 (3)	C3—C4—C21—C23	75.5 (4)
P1—C9—C10—C11	−179.3 (5)	C3—C4—C21—C24	−166.2 (4)
P1—C9—C14—C13	178.9 (4)	C5—C1—C2—P1	−179.7 (3)
P1—C15—C16—C17	179.1 (3)	C9—P1—C2—C1	178.4 (3)
P1—C15—C20—C19	−178.4 (3)	C9—P1—C3—C4	44.9 (3)
O1—C1—C2—P1	1.7 (5)	C9—P1—C15—C16	−112.2 (3)
O1—C1—C5—C6	−29.7 (6)	C9—P1—C15—C20	67.6 (4)
O1—C1—C5—C7	87.1 (5)	C9—C10—C11—C12	0.7 (10)
O1—C1—C5—C8	−155.1 (5)	C10—C9—C14—C13	0.6 (8)
O2—C4—C21—C22	136.3 (4)	C10—C11—C12—C13	0.0 (10)
O2—C4—C21—C23	−103.4 (5)	C11—C12—C13—C14	−0.5 (9)
O2—C4—C21—C24	14.9 (6)	C12—C13—C14—C9	0.2 (9)
C2—P1—C3—C4	159.8 (3)	C14—C9—C10—C11	−1.1 (8)
C2—P1—C9—C10	−51.0 (4)	C15—P1—C2—C1	−62.2 (3)
C2—P1—C9—C14	130.8 (4)	C15—P1—C3—C4	−78.0 (3)
C2—P1—C15—C16	130.6 (3)	C15—P1—C9—C10	−170.8 (4)
C2—P1—C15—C20	−49.7 (4)	C15—P1—C9—C14	11.0 (4)
C2—C1—C5—C6	151.7 (4)	C15—C16—C17—C18	−1.1 (7)
C2—C1—C5—C7	−91.5 (5)	C16—C15—C20—C19	1.3 (7)
C2—C1—C5—C8	26.3 (6)	C16—C17—C18—C19	2.1 (7)
C3—P1—C2—C1	61.5 (3)	C17—C18—C19—C20	−1.4 (7)
C3—P1—C9—C10	64.5 (4)	C18—C19—C20—C15	−0.3 (7)
C3—P1—C9—C14	−113.7 (4)	C20—C15—C16—C17	−0.7 (6)
C3—P1—C15—C16	10.3 (4)	Cl2A^i^—Cl2A—C1A—Cl1A	113.6 (8)
C3—P1—C15—C20	−169.9 (3)	Cl2A^i^—Cl2A—C1A—Cl3A	0.0 (11)

Symmetry code: (i) −*x*+1, *y*, −*z*+3/2.

### Hydrogen-bond geometry (Å, º)

**Table:**

*D*—H···*A*	*D*—H	H···*A*	*D*···*A*	*D*—H···*A*
C2—H2*A*···Br1*B*^ii^	0.99	2.85	3.840 (9)	174
C2—H2*A*···Br1*C*^ii^	0.99	2.75	3.712 (6)	164
C2—H2*A*···Br1*A*^ii^	0.99	2.95	3.941 (7)	177
C2—H2*B*···Br1*B*	0.99	2.95	3.767 (9)	141
C2—H2*B*···Br1*C*	0.99	2.97	3.717 (6)	133
C2—H2*B*···Br1*A*	0.99	2.97	3.831 (7)	145
C3—H3*B*···Br1*B*	0.99	2.78	3.740 (9)	163
C3—H3*B*···Br1*C*	0.99	2.70	3.644 (6)	159
C3—H3*B*···Br1*A*	0.99	2.86	3.816 (7)	164
C16—H16···O1^iii^	0.95	2.43	3.287 (5)	150
C1*A*—H1*A*···Br1*A*	1.00	2.59	3.527 (9)	156

Symmetry codes: (ii) −*x*+3/2, *y*+1/2, −*z*+3/2; (iii) −*x*+3/2, −*y*+1/2, −*z*+1.
