# Supplementary figures and images for: Crystal structure of bis­(3,3-dimethyl-2-oxobut­yl)di­phenyl­phospho­nium bromide chloro­form monosolvate
Source: Acta Crystallogr E Crystallogr Commun. 2015 Apr 25;71(Pt 5):o339–40. doi: 10.1107/S205698901500763X (PMC4420080; doi:10.1107/S205698901500763X)

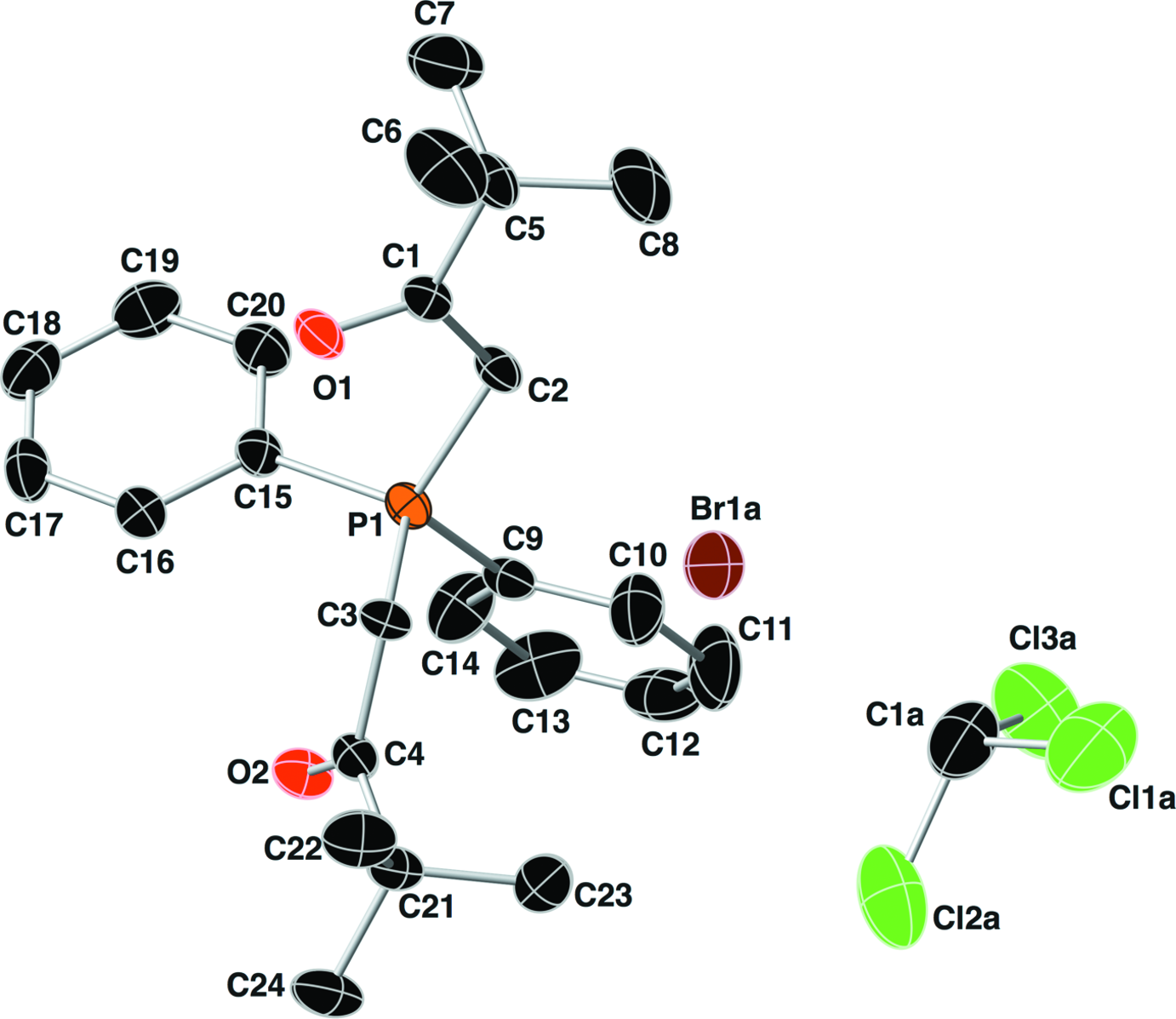

Supplement: Supplementary file 4 [file e-71-0o339-fig1.tif]

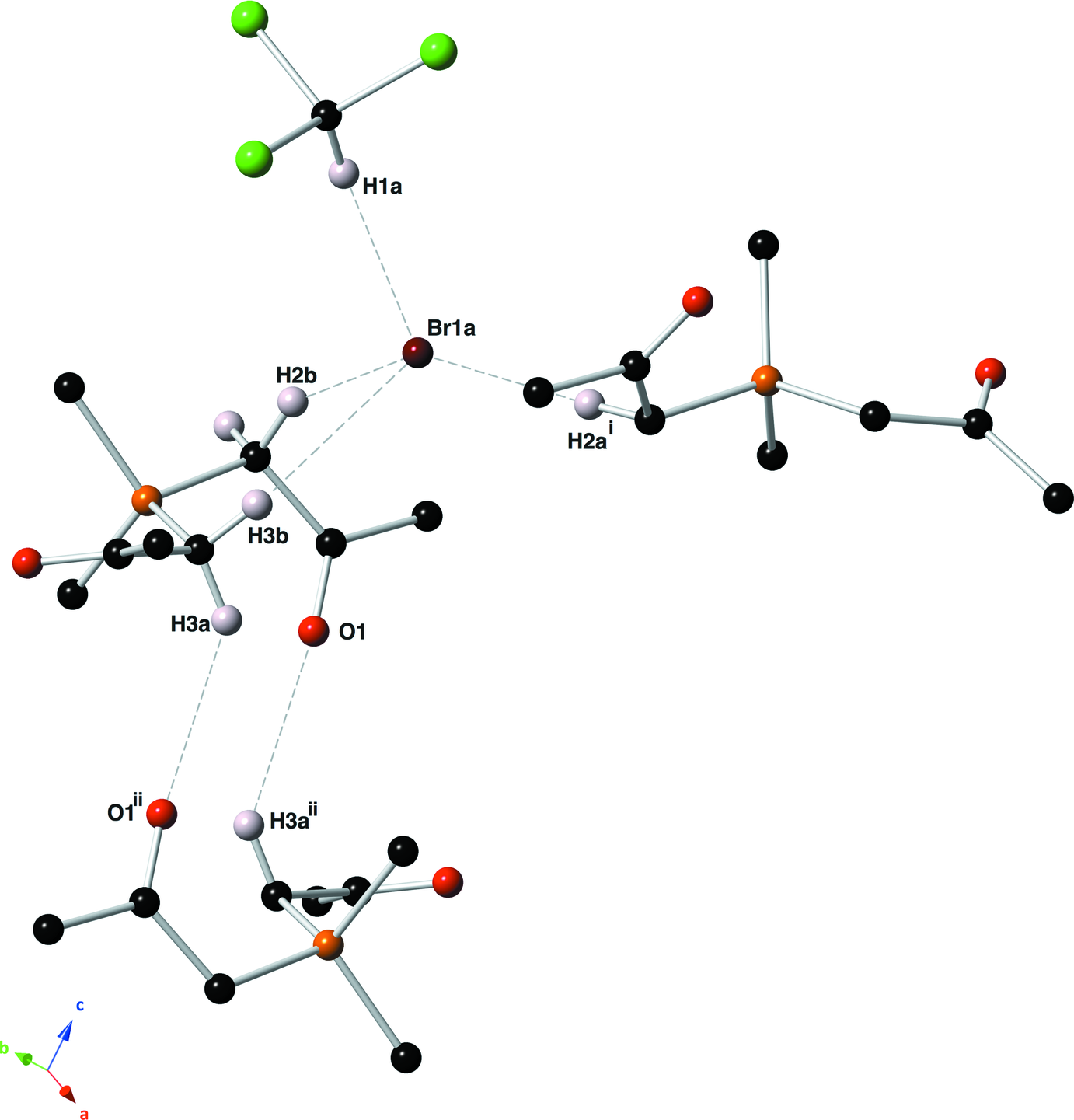

Supplement: Supplementary file 5 [file e-71-0o339-fig2.tif]
